# Increased risk of provisional premenstrual dysphoric disorder (PMDD) among females with attention-deficit hyperactivity disorder (ADHD): cross-sectional survey study

**DOI:** 10.1192/bjp.2025.104

**Published:** 2025-06

**Authors:** Thomas Broughton, Ellen Lambert, Jasmin Wertz, Jessica Agnew-Blais

**Affiliations:** Department of Psychology, School of Biological and Behavioural Sciences, Queen Mary University, UK; Institute of Psychiatry, Psychology and Neuroscience, King’s College London, UK; Department of Psychology, University of Edinburgh, UK

**Keywords:** Attention-deficit hyperactivity disorder (ADHD), premenstrual dysphoric disorder (PMDD), women’s health

## Abstract

**Background:**

Due to historical under-recognition of attention-deficit hyperactivity disorder (ADHD) among girls and women, little is known about female-specific factors that may affect individuals with ADHD, including those related to changes in ovarian hormones (e.g. across the menstrual cycle).

**Aims:**

We investigated whether females with a self-reported clinical diagnosis of ADHD are more likely to experience premenstrual dysphoric disorder (PMDD). We also examined associations between PMDD and ADHD defined by a symptom and impairment threshold.

**Method:**

Participants were aged between 18 and 34 years, were assigned female at birth and were recruited via Prolific.com (*n* = 715). Participants self-reported clinician diagnosis of ADHD, depression and anxiety. ADHD symptoms were assessed via the Adult ADHD Self-Report Scale (ASRS), to which we applied a DSM-5-based symptom and impairment cut-off (‘ASRS-based ADHD’). PMDD symptoms were assessed via the Premenstrual Symptoms Screening Tool (PSST), which identifies provisional PMDD. Using Poisson regression models, we compared risk for provisional PMDD among females with ADHD (self-reported clinical diagnosis [*n* = 102] or ASRS-based [*n* = 229]) with a non-ADHD reference group (*n* = 305). We additionally compared risk for provisional PMDD among individuals with ADHD and depression/anxiety diagnoses, ADHD only and a non-ADHD reference group.

**Results:**

The prevalence of provisional PMDD was elevated among individuals with a self-reported clinical ADHD diagnosis (31.4%), and among participants with ASRS-based ADHD (41.1%), compared with the non-ADHD reference group (9.8%). Individuals with ASRS-based ADHD and depression and/or anxiety diagnoses were at highest risk for provisional PMDD (relative risk 4.53 [3.10, 6.61]) compared with the non-ADHD reference group.

**Conclusions:**

Clinicians should be aware that individuals with a diagnosis of ADHD, or with high ADHD symptom levels, and who have a menstrual cycle may be more likely to experience PMDD. Future research should investigate the underlying mechanisms that link ADHD and disorders associated with hormonal sensitivity, such as PMDD.

Attention-deficit hyperactivity disorder (ADHD) is a condition characterised by impairing levels of inattentive, hyperactive and impulsive behaviours.^1^ ADHD has historically been considered a childhood-limited condition predominant in males, leading to an under-recognition of ADHD among girls and women. However, while ADHD diagnoses show a strong male predominance in childhood (∼3:1 male:female), by adulthood this ratio narrows to nearer 1.5:1.^2^ Females tend to be diagnosed with ADHD later in life, probably due, at least in part, to differences in ADHD symptom manifestation, because females may be more likely to exhibit inattentive symptoms and emotional impulsivity that may be less obvious to parents and teachers. Additionally, ADHD among females may be subject to diagnostic overshadowing by disorders that more commonly affect females, such as depression or anxiety.^3^ Due to the historical focus on males and ADHD, female-specific factors, including effects of changes in ovarian hormones across the menstrual cycle, have been under-researched in ADHD.

Premenstrual dysphoric disorder (PMDD) is a DSM-5 diagnosis characterised by impairing levels of affective lability, irritability, depressed mood and anxiety that onset in the week prior to menstruation and resolve in the days after menstruation begins. Additional symptoms may include decreased interest in usual activities, difficulty concentrating, fatigue, sleep and/or appetite changes and physical symptoms.^1^ Because a clinical diagnosis of PMDD requires 2 months of prospective symptom tracking, cross-sectional assessment is therefore considered a provisional diagnosis. A recent meta-analysis identified rates of confirmed clinically diagnosed PMDD of 3.2% and of provisional PMDD of 7.7%.^4^ PMDD has been associated with severe outcomes, including increased suicidality.^5,6^

## ADHD and hormonal sensitivity

Two recent studies suggest that individuals with ADHD may be at greater risk for mental health problems associated with times of hormonal change, including an increased risk for postpartum depression and depression following initiation of hormonal contraceptives.^7,8^ Furthermore, one study to date has investigated PMDD among women in out-patient treatment for ADHD and found that 45.5% met criteria for provisional PMDD, compared with 28.7% in a general population survey.^9^ However, questions remain regarding whether higher rates of PMDD are limited to individuals in clinical care for ADHD or are also elevated in the general population with ADHD, and about the role of comorbid depression and anxiety in the association between ADHD and PMDD.

In the current study we investigated risk for provisional PMDD among females with a self-reported clinical diagnosis of ADHD from a non-clinical, population-based sample. Additionally, because clinical diagnosis might not capture the entire ADHD population, especially among females who may go underdiagnosed due to clinicians requiring evidence for their (less obvious) childhood symptoms, and because of long waiting lists for adult ADHD assessment,^10^ we also examined the risk for PMDD among individuals by applying a DSM-5 ADHD symptom and impairment cut-off. To investigate the role of depression and anxiety, we assessed rates of PMDD among individuals with ADHD as well as depression and/or anxiety, because both ADHD and PMDD are associated with increased risk for depression and anxiety.^11–13^

## Method

### Study population

We recruited participants using Prolific (www.prolific.com), an online platform for research study participant recruitment. On Prolific, potential participants can select from a range of demographic, health and other characteristics corresponding to potential study eligibility criteria, so that they are offered participation in studies for which they are eligible. To recruit a sufficient number of females with ADHD, we used this ‘prescreen’ feature and recruited participants in two waves. First, we recruited 370 females who endorsed the prescreen question ‘Do you consider yourself to have attention deficit disorder (ADD)/attention deficit hyperactivity disorder (ADHD)?’. Second, we recruited 370 females who responded negatively to this prescreen question. Therefore, this study over-recruited for participants with self-reported ADHD, and the overall prevalence of ADHD is not representative of the general population. Supplementary Table 1 provides demographic and clinical information on the prescreened groups. The authors assert that all procedures contributing to this work comply with the ethical standards of the relevant national and institutional committees on human experimentation, and with the Helsinki Declaration of 1975 as revised in 2013. All procedures involving human subjects/patients were approved by the Queen Mary University London Ethics of Research Committee (approval no. QMERC22.377), and all participants gave written informed consent. Participants were compensated at a rate of £10/h, with completion of the study estimated to take a maximum of 20 min. Recruitment took place on 26–27 September 2023.

Our study included participants assigned female at birth, because we are investigating a disorder linked to the menstrual cycle. Participants under the age of 18 and over the age of 34 years were excluded to reflect females who are more likely to have regular menstrual cycles. All participants were residents of the UK. Individuals were excluded if they did not meet eligibility criteria (*n* = 9) or provided incomplete or invalid data (*n* = 16) (Supplementary Table 2). After exclusions, 715 females were included.

### Measures

#### Self-reported clinical ADHD diagnosis

**Fig. 1:**
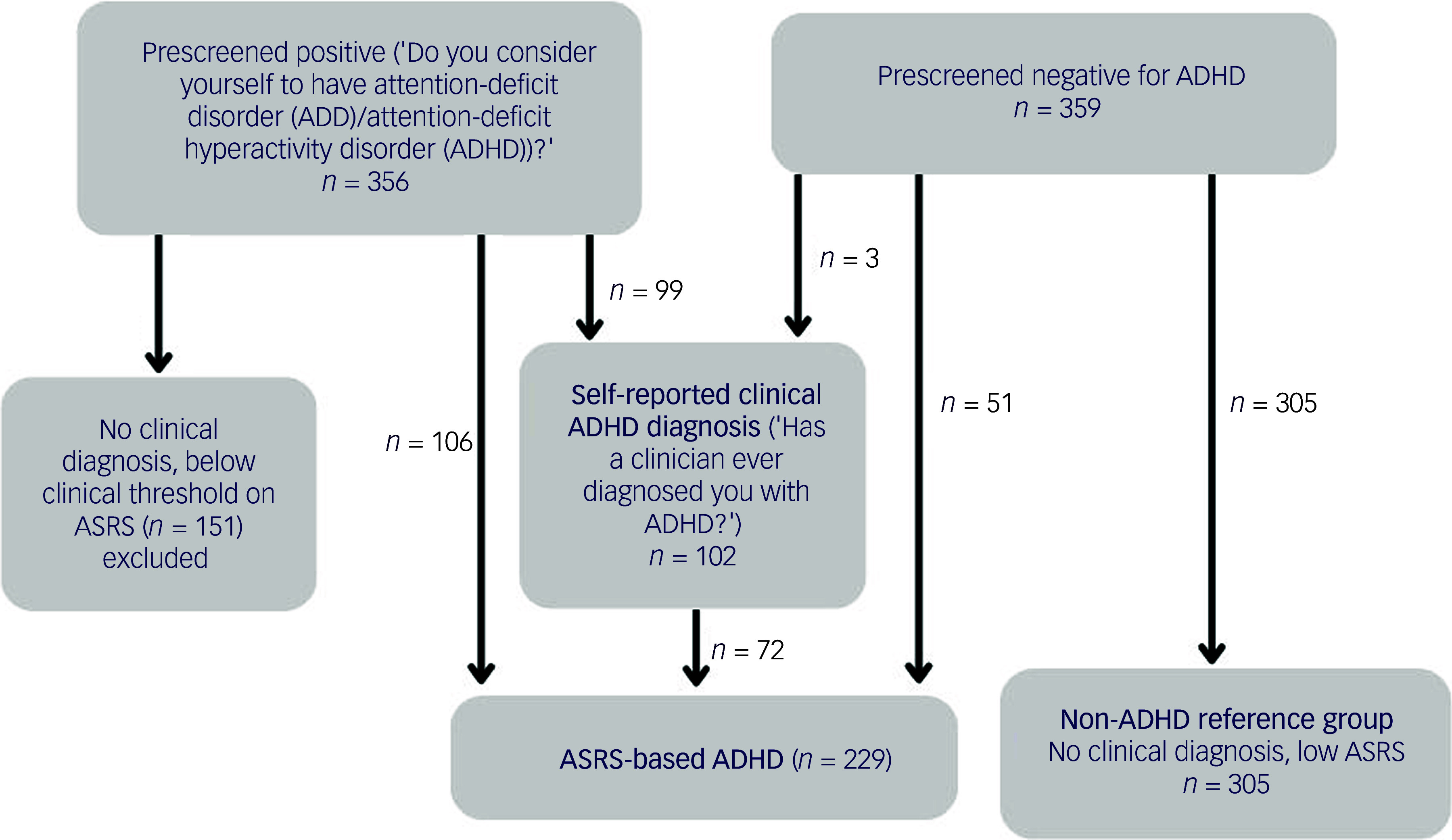
Recruitment of participants prescreened positive or negative and subsequent classification as self-reported clinical ADHD, ASRS-based ADHD or non-ADHD reference group. Note: participants were excluded due to being either ineligible (*n* = 9) or having incomplete or invalid data (*n* = 16) from the original *n* = 370 per recruited group; details provided in Supplementary Table 2. ADHD, attention-deficit hyperactivity disorder; ASRS, Adult ADHD Self-Report Scale.

Self-reported clinical diagnosis of ADHD was defined by participants endorsing the question ‘Has a clinician ever diagnosed you with ADHD?’. Among individuals who prescreened positive for ADHD (i.e. considered themselves to have ADHD on the prescreening question), 27.8% reported receiving a clinical diagnosis of ADHD (Fig. 1).

#### ASRS-based ADHD

The Adult ADHD Self-Report Scale (ASRS) was used to assess ADHD symptoms (ASRS-v1.1)^14^ and define an ‘ASRS-based’ ADHD group. The ASRS includes 18 items based on DSM-IV-TR ADHD; population surveys report that the ASRS has moderate sensitivity (68.7%) and high specificity (99.5%^14^). In addition to ADHD symptoms, participants were asked whether they had experienced impairment across three settings (’in your home life’, ‘at work or in school’ and ‘in your social interactions with others’). ASRS-based ADHD was defined as (a) falling above the DSM cut-off of five or more symptoms and (b) endorsing interference with ability to function ‘often’ or ‘always’ in at least two of three settings. Among individuals who prescreened positive for ADHD (i.e. considered themselves to have ADHD), 49.4% met ASRS-based criteria; among individuals who did not prescreen positive for ADHD (i.e. did not consider themselves to have ADHD), 14.2% met ASRS-based criteria (Fig. 1).

The non-ADHD reference group was defined as those who did not consider themselves to have ADHD in the prescreen question, did not self-report a clinical diagnosis of ADHD and did not meet ASRS-based ADHD criteria (*n* = 305). Individuals who endorsed the prescreen question of considering themselves to have ADHD, but who did not go on to self-report a clinical ADHD diagnosis or meet ASRS-based ADHD criteria, were excluded, because their ADHD versus non-ADHD reference group status was unclear (*n* = 151). Figure 1 illustrates the different ADHD, reference and excluded participant groups; 70.6% of those with a self-reported ADHD clinical diagnosis met ASRS-based criteria, and 31.4% of those meeting ASRS-based criteria self-reported an ADHD diagnosis.

#### Depression/anxiety

We also queried whether participants had depression and/or anxiety with the following questions: ‘Has a clinician ever diagnosed you with depression’ and ‘Has a clinician ever diagnosed you with anxiety’. We then created groups to reflect those with ADHD and depression and/or anxiety diagnoses: individuals with ADHD and anxiety/depression diagnoses (self-reported diagnosis, *n* = 77; ASRS-based, *n* = 173), individuals with ADHD only (no depression or anxiety diagnoses; self-reported ADHD diagnosis, *n* = 25; ASRS-based, *n* = 56) and those in the non-ADHD reference group described above (*n* = 305). Supplementary Table 3 provides demographic and clinical characteristics for these groups.

#### Provisional PMDD

We used the Premenstrual Symptoms Screening Tool (PSST) to assess premenstrual symptoms.^15^ Given the cross-sectional nature of this study, we used the PSST as it is a rapid, accessible and useful tool for assessing PMDD symptoms. Measurement of PMDD symptoms based on recall, as with the PSST, cannot be used to formally diagnose PMDD because diagnosis requires 2 months of prospective symptom recording. However, the PSST is nevertheless designed to identify people who experience clinically significant symptoms.^15^ The PSST operationalises DSM-IV-TR criteria for PMDD and queries whether females experience symptoms that start in the days prior to their period and stop within a few days of bleeding, including the following: anger/irritability; anxiety/tension; tearfulness/rejection sensitivity; depressed mood; decreased interest in work, home or social activities; difficulty concentrating; fatigue; overeating; insomnia; hypersomnia; overwhelm; and physical symptoms. Provisional PMDD criteria included (a) at least one of the symptoms: anger/irritability, anxiety/tension, tearful/increased sensitivity to rejection and depressed mood/hopelessness, rated as severe; (b) at least four other symptoms rated as moderate to severe; and (c) impairment rated as severe in at least one setting. Supplementary Methods includes additional information on the PSST (Supplementary Appendix 1).

#### Other measures

We collected demographic information, including age and educational attainment, as well as information on whether respondents were currently using hormonal contraceptives and, if so, what type. We also queried whether participants were currently taking, or had ever taken, ADHD medication (questions are included in Supplementary Methods, Appendix 2).

### Statistical analysis

We compared self-reported clinical ADHD and ASRS-based ADHD groups with the non-ADHD reference group on demographic and mental health characteristics using chi-square and *t*-tests (Table 1).

**Table 1:** Sociodemographic and clinical characteristics of participants with a self-reported clinical ADHD diagnosis, ASRS-based ADHD and non-ADHD reference group

Characteristic	Self-reported clinical ADHD diagnosis (*n* = 102)	ASRS-based ADHD (*n* = 229)	Non-ADHD reference group (*n* = 305)	Statistical comparisons	
				Clinical diagnosis versus ref.	ASRS-based versus ref.
Age (years), mean (s.d.)	27.74 (3.93)	27.93 (3.94)	28.00 (4.03)	*t*(406) = −0.58	*t*(533) = −0.19
Education, *n* (%)				*χ* ^2^(3) = 2.41	*χ* ^2^(3) = 5.80
Secondary school	29 (28.43)	64 (27.95)	108 (35.41)		
Bachelor’s degree	41 (40.20)	97 (42.36)	116 (38.03)		
Master’s degree/PhD	25 (24.51)	50 (21.83)	68 (22.30)		
Other	7 (6.86)	18 (7.86)	13 (4.26)		
Hormonal contraceptive use, *n* (%)	43 (42.16)	86 (37.55)	122 (40.00)	*χ* ^2^(1) = 0.15	*χ* ^2^(1) = 0.33
Currently taking ADHD medication, *n* (%)	39 (38.24)	29 (12.66)	0	–	–
Ever taken ADHD medication, *n* (%)	65 (63.73)	46 (20.09)	0	–	–
ASRS total score (0–18), mean (s.d.)	13.55 (3.19)	13.26 (2.93)	4.52 (3.52)		
Self-reported clinical depression diagnosis, *n* (%)	62 (60.78)	162 (70.74)	78 (25.57)	*χ* ^2^(1) = 42.00***	*χ* ^2^(1) = 68.47***
Self-reported clinical anxiety diagnosis, *n* (%)	68 (66.67)	140 (61.14)	98 (32.13)	*χ* ^2^ (1) = 37.75***	*χ* ^2^(1) = 78.05***
Self-reported clinical ADHD diagnosis, *n* (%)	102 (100)	72 (31.44)	0	–	–
Met ASRS cut-off ADHD, *n* (%)	72 (70.59)	229 (100)	0	–	–
Provisional PMDD, *n* (%)	32 (31.37)	94 (41.05)	30 (9.84)	*χ* ^2^(1) = 27.46***	*χ* ^2^(1) = 71.47***

ADHD, attention-deficit hyperactivity disorder; ASRS, Adult ADHD Self-Report Scale; PMDD, premenstrual dysphoric disorder; ***P < 0.001.

To understand whether self-reported ADHD diagnosis was associated with provisional PMDD, we assessed the prevalence of provisional PMDD amongst those with a self-reported ADHD diagnosis compared with the non-ADHD reference group. Next, we calculated the relative risk for provisional PMDD associated with having a self-reported clinical diagnosis of ADHD via a Poisson regression with a robust error variance, because this type of regression better approximates the relative risk when the outcome is not rare.^16^ We took a similar approach to comparing those with ASRS-based ADHD with the non-ADHD reference group, calculating the prevalence in each group and the relative risk using Poisson regression.

To assess the contribution of additional diagnoses of anxiety and/or depression to the association between ADHD and provisional PMDD, we further compared risk for provisional PMDD among the following: (a) individuals with ADHD (self-reported and ASRS-based) and anxiety/depression diagnoses and (b) individuals with ADHD only (no anxiety or depression diagnosis) with the non-ADHD reference group, using Poisson regression.

Furthermore, to understand whether people with ADHD and PMDD show a different profile of PMDD symptoms, we calculated the prevalence of each symptom rated as moderate/severe on the PSST across the ADHD and non-ADHD reference groups among those with provisional PMDD. In sensitivity analyses we compared the rates of ADHD, PMDD and depression/anxiety among females taking or not taking hormonal contraceptives. All analyses were conducted using STATA v16.1.

## Results

Participants who reported a clinical ADHD diagnosis (*n* = 102) or met ASRS-based ADHD criteria (*n* = 229) did not significantly differ from the non-ADHD reference group (*n* = 305) on age, educational attainment or hormonal contraceptive use (Table 1). Participants with a self-reported clinical ADHD diagnosis had higher rates of depression and anxiety diagnoses (60.8 and 66.7%, respectively), as well as higher mean ASRS scores (mean = 13.5, s.d. = 3.2) compared with the non-ADHD reference group (mean = 4.5, s.d. = 3.5). Participants with ASRS-based ADHD also had higher rates of depression and anxiety diagnoses and higher ASRS scores (mean = 13.3, s.d. = 2.9) compared with the non-ADHD reference group.

### ADHD and provisional PMDD

Participants with a self-reported clinical diagnosis of ADHD were more likely to report symptoms consistent with provisional PMDD (31.4%) compared with the non-ADHD reference group (9.8%), corresponding to a 3.19-fold higher risk of provisional PMDD (95% CI: [2.04, 4.98], *P* < 0.001). Participants meeting ASRS-based ADHD criteria were more likely to report symptoms consistent with provisional PMDD (41.1%) compared with those who did not have a diagnosis of ADHD, corresponding to a 4.17-fold higher risk of provisional PMDD (95% CI: [2.87, 6.07], *P* < 0.001).

### ADHD, depression, anxiety and PMDD

**Fig. 2:**
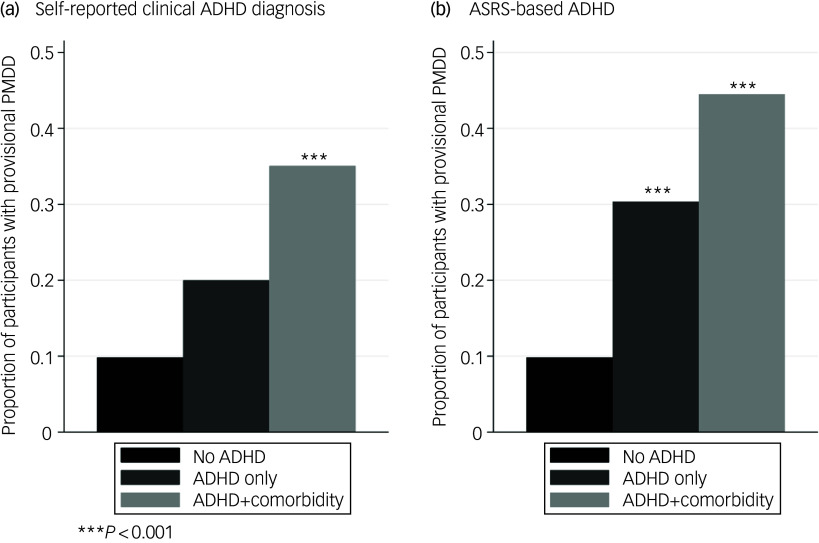
Prevalence of provisional premenstrual dysphoric disorder (PMDD) comparing participants in the non-ADHD reference group (*n* = 305) to those with ADHD only and ADHD with self-reported depression and/or anxiety diagnoses ((a) with self-reported clinical diagnoses, ADHD + comorbidity, *n* = 77; ADHD only, *n* = 25), and (b) with ASRS-based ADHD, ADHD + comorbidity, *n* = 173; ADHD only, *n* = 56). PMDD, premenstrual dysphoric disorder; ADHD, attention-deficit hyperactivity disorder; ASRS, Adult ADHD Self-Report Scale.

Individuals with self-reported diagnoses of ADHD and anxiety and/or depression had the highest prevalence of provisional PMDD (35.1%), while the self-reported ADHD diagnosis-only group had a prevalence of 20.0% (*n* = 25) and the non-ADHD reference group of 9.8% (*n* = 305) (Fig. 2(a)). Individuals with a diagnosis of ADHD and anxiety and/or depression had higher risk for PMDD compared with those without any of these diagnoses (relative risk equals 3.56 [2.26, 5.63], *P* < 0.001). Individuals with an ADHD diagnosis only were at twice the risk of provisional PMDD compared with those without this diagnosis, although this did not reach statistical significance (relative risk equals 2.03 [0.86, 4.78], *P* = 0.10). Among those with ASRS-based ADHD there was a similar pattern of findings: those with ADHD and depression/anxiety had the greatest risk of provisional PMDD (relative risk equals 4.53 [3.10, 6.61], *P* < 0.001) (Fig. 2(b)). In this case the ADHD-only group was also significantly more likely to meet provisional PMDD criteria than the non-ADHD reference group (relative risk equals 3.09 [1.83, 5.21], *P* < 0.001).

### ADHD and individual PMDD symptoms

**Fig. 3:**
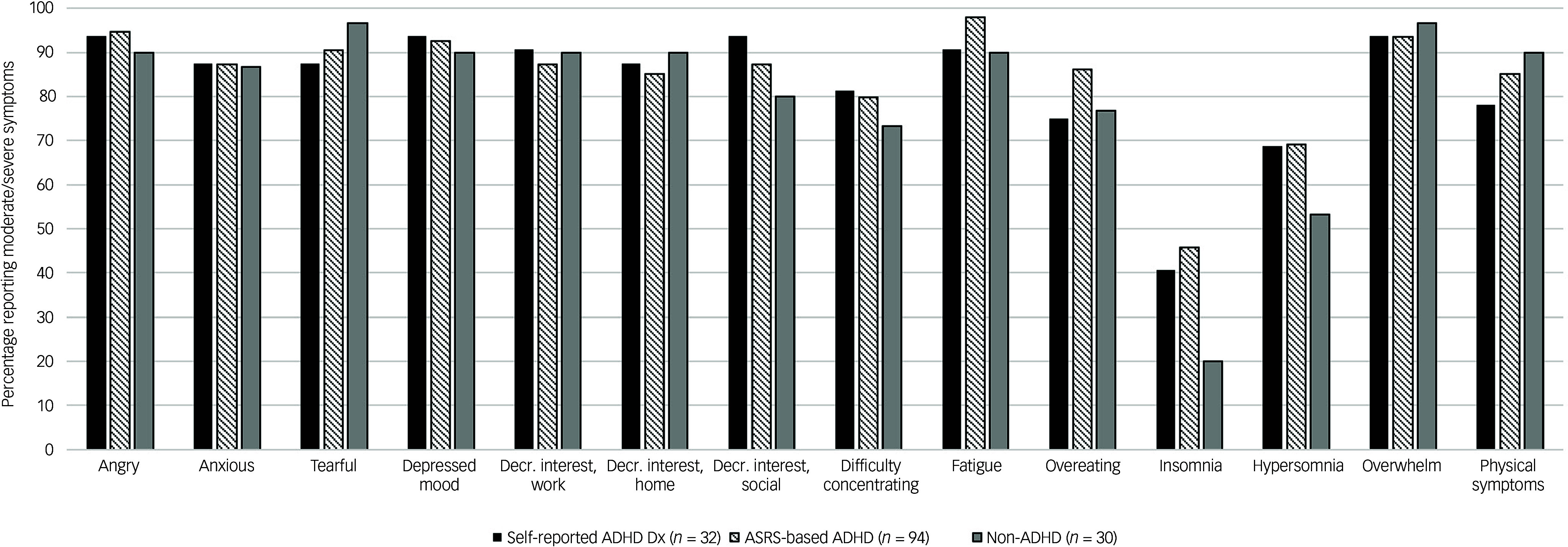
Percent reporting moderate/severe PMDD symptoms among the self-reported ADHD diagnosis, ASRS-based ADHD and non-ADHD reference groups among those meeting criteria for provisional PMDD. PMDD, premenstrual dysphoric disorder; ADHD, attention-deficit hyperactivity disorder; ASRS, Adult ADHD Self-Report Scale; Decr., decreased; Dx, diagnosis.

Among individuals with provisional PMDD, those with a self-reported ADHD diagnosis, ASRS-based ADHD and the non-ADHD reference group endorsed similar symptoms of PMDD most frequently, including feelings of anger, overwhelm, tearfulness and depressed mood (Fig. 3). Notably, the ADHD groups were more likely to endorse insomnia compared with the non-ADHD reference group.

### Sensitivity analyses

We found that rates of self-reported diagnoses of ADHD, depression, anxiety and provisional PMDD were similar for females who were taking a hormonal contraceptive versus those who were not (Supplementary Table 4). The magnitude of the association between self-reported clinical ADHD diagnosis and provisional PMDD was similar between females who were taking (relative risk 3.65 [2.05, 6.50]) and not taking (relative risk 2.62 [1.29, 5.30]) a hormonal contraceptive.

## Discussion

Although there has recently been a greater awareness of ADHD in females, research on how ADHD is associated with female-specific factors, such as those linked to the menstrual cycle, is lacking. In a survey of 715 females who self-reported or met criteria for a diagnosis of ADHD and those who did not, the former had higher rates of provisional PMDD. This was evident both for self-reported diagnosis and when using a well validated scale to identify those with ADHD. Individuals with ADHD and depression and/or anxiety diagnoses showed the highest prevalence of provisional PMDD.

### Risk of provisional PMDD is elevated among females with ADHD

The finding of a higher risk for PMDD among individuals with ADHD is consistent with a previous study^9^ that found that females with ADHD were more likely to experience provisional PMDD, assessed via the MINI Neuropsychiatric Interview Plus. Compared with the rates of provisional PMDD reported in this previous study (45.5%), we identified a slightly lower overall prevalence, ranging from 31.4% of participants with a self-reported clinical diagnosis of ADHD to 41.1% among participants meeting the ASRS-based ADHD criteria. This discrepancy may be due to differences in participant recruitment, because Dorani et al included participants from outpatient clinics and who may experience more severe ADHD compared with our population-based sample. However, despite distinct study populations, both studies observed an increased risk for provisional PMDD among females with ADHD. Additional evidence for a link between ADHD and PMDD is provided by a recent case-control study in Taiwan, which reported higher rates of ADHD among 58 women with a provisional PMDD diagnosis compared with 50 women without PMDD.^17^

Why females with ADHD experience higher risk for PMDD is a critical area for future investigation. Individual sensitivity to hormonal fluctuations may be an underpinning mechanism of the association between ADHD and premenstrual problems.^18^ Recent research suggests individuals with ADHD may be at higher risk for poor outcomes associated with times of hormonal change. A Swedish national registry study found that females with ADHD had a sixfold higher risk of depression associated with initiation of oral combined hormonal contraceptives compared with females without ADHD.^7^ Another registry study found that, during the postpartum period, females with a diagnosis of ADHD were approximately fivefold more likely to receive diagnoses of depression and anxiety disorders compared with those without an ADHD diagnosis.^8^

Evidence suggests that PMDD is not a result of abnormal levels of hormones, but of individual sensitivity to normative hormonal fluctuations.^19,20^ Greater sensitivity to these fluctuations among individuals with ADHD may be due to existing vulnerabilities related to neurotransmitters such as dopamine. The drop in oestrogen associated with the premenstrual period is associated with decreased dopamine availability: because dopamine plays a key role in ADHD, this change may be more likely to affect females with ADHD.^18,21,22^ Our findings that females with ADHD have an elevated risk for provisional PMDD are consistent with research examining other times of hormonal change, such as the postpartum period,^7,8,22^ suggesting that risk for females with ADHD is not specific to PMDD but may be more generally associated with fluctuations in ovarian hormone levels. It is crucial to consider the impact for females with ADHD of times of hormonal change, such as puberty, pregnancy, perimenopause and menopause.

### ASRS-based ADHD is also associated with increased provisional PMDD risk

While individuals who receive a clinical diagnosis of a mental health condition are generally more severely affected than those identified in population surveys,^23^ we additionally observed a higher risk for PMDD among a group identified by a well-validated ADHD scale. Thus, examining rates of PMDD only among those individuals with a clinical diagnosis of ADHD may underestimate the association of the two conditions. Our results suggest that clinicians assessing PMDD risk should account for high levels of symptoms and impairment even in the absence of a formal ADHD diagnosis, probably especially salient for individuals living in areas with long waiting lists for ADHD assessment.

### Individuals with ADHD and comorbid mental health problems have especially elevated provisional PMDD risk

Individuals with depression and anxiety have been found to be at increased risk for PMDD.^24,25^ Our findings add to this literature by showing that women with ADHD (both self-reported diagnosis and questionnaire-based) and additional diagnoses of depression or anxiety are at notably increased risk of provisional PMDD. ADHD is frequently comorbid with other mental health conditions,^7^ and our results underscore the importance of considering comorbidity and suggest that individuals with ADHD and additional mental health conditions may be a key population for PMDD screening.

### PMDD symptom profiles are similar between ADHD and non-ADHD groups

The overall profile of PMDD symptoms among individuals meeting provisional PMDD criteria was similar across those with ADHD (both clinical diagnosis and ASRS-based) and those without. Insomnia was the least commonly endorsed symptom, aligning with previous research,^15^ although those with ADHD and PMDD showed about twice the rate of endorsement of this symptom compared with those with no ADHD, suggesting there might be some specificity around sleep problems among people with ADHD and PMDD. The PMDD symptom of ‘difficulty concentrating’ was similarly endorsed among ADHD and non-ADHD groups, suggesting inattention symptoms related to PMDD were not specific to individuals with ADHD.

### Strengths and limitations

This study has several strengths, including investigating rates of provisional PMDD among females with ADHD in a population-based sample, rather than among individuals receiving clinical care. This is a strength because there may be selection biases associated with clinical populations (e.g. related to severity or access to care) that can lead to inflation in estimates of comorbidity.^26^ Additionally, we considered the role of comorbidity with depression/anxiety with risk for provisional PMDD. Moreover, administering the ASRS allowed us to investigate provisional PMDD among individuals with ADHD using DSM-5-based symptom and impairment cut-offs, thereby capturing a broader ADHD population.

However, there are also limitations. First, because this study was cross-sectional we were not able to apply the gold standard PMDD diagnosis, which requires daily reporting of symptoms over at least two menstrual cycles. Retrospective reporting of PMDD is associated with false positives compared with gold standard prospective ratings,^4,27–29^ so it is possible that our case group includes false positives for PMDD. However, subthreshold PMDD symptoms are still likely to cause distress and impairment.^15,30^ Additionally, given that little research has investigated PMDD among individuals with ADHD, our aim is that these initial findings will support further research that can incorporate prospective assessment of PMDD symptoms.

Additionally, because this study was cross-sectional we were unable to explicitly disentangle PMDD from premenstrual exacerbation (PME) of ADHD, depression or anxiety.^31^ Therefore, what may appear as provisional PMDD in our study may reflect exacerbations of other mental health conditions. More detailed prospective assessments in future studies can identify more clearly the asymptomatic window, in which symptoms are minimal or absent following menses, that can distinguish between PMDD and PME.

Our study population was recruited from an online research platform and thus may not be demographically representative of the general population (evident, for example, in the relatively high educational level of the sample; Table 1). Additionally, while the study was not advertised as assessing PMDD specifically, the reference to mood changes during the menstrual cycle (to transparently describe the study to potential participants) may have attracted individuals who felt this was relevant to them. While we included a quantitative measure of ADHD, we queried only the clinical diagnosis of depression and anxiety and thus we cannot examine the quantitative effects of the symptoms of these disorders on PMDD risk. Additionally, we did not collect data on medications for other disorders such as depression or anxiety, which may affect PMDD symptoms.^32^ While we excluded participants who likely did not have menstrual cycles due to pregnancy, breastfeeding or menopause, we did not ask participants whether they had regular menstrual cycles and it is possible that some participants had irregular or anovulatory menstrual cycles. Future research could use a measure such as the Reproductive Status Questionnaire to gather more information regarding the menstrual cycle.^33^ Lastly, because we did not query age of diagnosis of ADHD, some individuals who self-reported a clinical ADHD diagnosis may have had ADHD in childhood but no longer meet diagnostic criteria. However, research finds that many individuals with ADHD continue to experience impairing symptoms into adulthood, and rates of ADHD persistence in girls in clinical cohorts are high.^34,35^ Nevertheless, the fact that some individuals in the self-reported clinical diagnosis group may no longer have met ADHD criteria might explain why we find stronger associations with PMDD in the ASRS-based group, which may better reflect current ADHD.

Despite these limitations, our study addresses two understudied conditions in a population-based cohort, providing valuable information on increased risk for provisional PMDD among females with ADHD. Future research should gather more detailed information on PMDD and ADHD from larger cohorts that are more representative of the general population (especially with regard to educational attainment), with prospective data collection over the course of at least two menstrual cycles; it should also consider the potential modifying effects of psychiatric and hormonal medications. This research should adhere to transdiagnostic frameworks such as the recent Dimensional Affective Sensitivity to Hormones across the Menstrual Cycle (DASH-MC), which aims to refine research on the influence of ovarian hormone fluctuations on risk of psychopathology.^36^

### Clinical implications

Clinicians and other healthcare professionals should be aware of the possibility that females with a diagnosis, or high level of symptoms, of ADHD may be at increased risk for PMDD, premenstrual problems or premenstrual exacerbation of symptoms. Current first-line treatment options for premenstrual problems include antidepressant medications, hormonal contraceptives and cognitive behavioural therapy.^37,38^ It is not known whether these treatments differ in their effectiveness among individuals with ADHD. A recent case series in which psychostimulant dosage was increased during the premenstrual stage in nine individuals with ADHD and co-occurring PMDD found improvement in ADHD and mood symptoms.^39^ Developing efficacious and appropriate treatment pathways that account for both ADHD symptoms and individual sensitivity to hormonal fluctuations is a crucial area for future research.^8,9^

Females with ADHD may be more vulnerable to conditions related to hormonal sensitivity throughout their lifespan, including PMDD. PMDD is a serious mental health condition and clinicians should consider screening for PMDD among females with an ADHD diagnosis or high ADHD symptom levels. It is crucial to establish mechanisms underpinning the relationship between ADHD and sensitivities to hormonal fluctuations across the lifespan, such as during puberty, the menstrual cycle, menopause and the perinatal period. A better understanding of the link between ADHD and times of hormonal changes may reduce health inequalities and diagnostic bias in females with ADHD.

## Supporting information

Broughton et al. supplementary material 1 Broughton et al. supplementary material

Broughton et al. supplementary material 2 Broughton et al. supplementary material

## Data Availability

De-identified research data to be shared with independent researchers can be provided on reasonable request to J.A.-.B. Code and materials for all analyses were curated and written by T.B. and J.A.-B., and are available on reasonable request to J.A.-B. The PSST, authored by Steiner et al, is the copyright of McMaster University (Copyright ©2003, McMaster University). PSST has been provided under licence from McMaster University and must not be copied, distributed or used in any way without the prior written consent of McMaster University. For licensing details, contact the McMaster Industry Liaison Office at McMaster University

## Supplementary material

The supplementary material can be found online at https://doi.org/10.1192/bjp.2025.104
