# Supplementary material for: Increased risk of provisional premenstrual dysphoric disorder (PMDD) among females with attention-deficit hyperactivity disorder (ADHD): cross-sectional survey study
Source: Br J Psychiatry. 2025 Jun 18;226(6):410–7. doi: 10.1192/bjp.2025.104 (PMC7617793; doi:10.1192/bjp.2025.104)
Supplement: Broughton et al. supplementary material 1 — Broughton et al. supplementary material [file S0007125025001047sup001.docx]

**Appendix S1: Questionnaire Appendix**

Demographic Questions

What is your age? ____

What is the highest level of education you’ve attained?

- Secondary school^a^
- Bachelor’s degree
- Master’s degree
- PhD
- Other (please specify________)

Other questions

Are you currently pregnant?

- Yes
- No

Are you currently breastfeeding?

- Yes
- No

Medication Questions

Do you take any medication for ADHD?

If so, what medication ______________

Have you ever taken any medication for ADHD?

Do you use any of the following contraceptives?

- Birth control pills
- The hormonal coil (or hormonal IUD; e.g. Mirena coil)^b^
- The copper coil (or non-hormonal IUD) ^b^
- Other (please specify________)

Has a clinician ever diagnosed you with any of the following:

- ADHD
- Depression
- Anxiety
- None of the above

Appendix S2 – Further questionnaire information

Adult ADHD Self-Report Scale (ASRS)

The ASRS includes eighteen items based on DSM-IV-TR ADHD criteria rated on a five-point scale (“Never”, “Rarely” “Sometimes”, “Often”, “Very Often”); an item was considered to be positively endorsed based on recommended cut-offs. Population surveys have shown that the ASRS has a moderate sensitivity (68.7%), high specificity (99.5%(1)), high internal consistency(2) and good test-retest reliability.(3)

Premenstrual symptoms screening tool (PSST)

The PSST operationalises DSM-IV-TR criteria for PMDD and queries whether females experience symptoms that start in the days prior to their period and stop within a few days of bleeding (4).  Fourteen items related to premenstrual symptoms (as highlighted in the introduction) are included. The impact of symptoms on functioning in work/school, social, and family settings was also measured; each item is rated on a four-point scale (‘Not at all’, ‘Mild’, ‘Moderate’, ‘Severe’).  In order to meet criteria for provisional PMDD: (a) at least one of the first four items (‘Anger/irritability’; ‘Anxiety/tension’ ‘Tearful/increased sensitivity to rejection’; ‘Depressed mood/hopelessness’) must be rated as severe, (b) at least four of the other fourteen items must be rated as moderate to severe, and (c) impairment must be rated as severe in at least one setting.

 Refences

1. Kessler RC, Adler L, Ames M, Demler O, Faraone S, Hiripi E, et al. The World Health Organization Adult ADHD Self-Report Scale (ASRS): a short screening scale for use in the general population. Psychol Med. 2005;35(2):245-56.

2. Adler LA, Spencer T, Faraone SV, Kessler RC, Howes MJ, Biederman J, Secnik K. Validity of pilot Adult ADHD Self- Report Scale (ASRS) to Rate Adult ADHD symptoms. Ann Clin Psychiatry. 2006;18(3):145-8.

3. Matza LS, Van Brunt DL, Cates C, Murray LT. Test-retest reliability of two patient-report measures for use in adults with ADHD. J Atten Disord. 2011;15(7):557-63.

4. Steiner M, Macdougall M, Brown E. The premenstrual symptoms screening tool (PSST) for clinicians. Arch Womens Ment Health. 2003;6(3):203-9.
