# Supplementary material for: Increased risk of provisional premenstrual dysphoric disorder (PMDD) among females with attention-deficit hyperactivity disorder (ADHD): cross-sectional survey study
Source: Br J Psychiatry. 2025 Jun 18;226(6):410–7. doi: 10.1192/bjp.2025.104 (PMC7617793; doi:10.1192/bjp.2025.104)
Supplement: Broughton et al. supplementary material 2 — Broughton et al. supplementary material [file S0007125025001047sup002.docx]

Supplemental Table 1: Sociodemographic and clinical characteristics of individuals who prescreened positively for ADHD and negatively for ADHD included in the current study

|  | Prescreened negative for ADHD (N=359) | Prescreened positive for ADHD (N=356) |
| --- | --- | --- |
| Age, mean (SD) | 28.04 (3.96) | 27.75 (3.96) |
| Education, N (%) |  |  |
| Secondary | 123 (34.26) | 99 (27.81) |
| Bachelor's degree | 138 (38.44) | 154 (43.26) |
| Master's degree/PhD | 82 (22.84) | 77 (21.63) |
| Other | 16 (4.46) | 26 (7.30) |
| Hormonal contraceptive use, N (%) | 144 (40.11) | 138 (38.76) |
| Currently taking ADHD medication, N (%) | - | 39 (10.96) |
| Ever taken ADHD medication, N (%) | 3 (0.84) | 62 (17.42) |
| ASRS total score (0-18), M (SD) | 5.65 (4.40) | 11.50 (4.00) |
| Depression diagnosis, N, (%) | 107 (29.81) | 190 (53.37) |
| Anxiety diagnosis, N (%) | 134 (37.33) | 213 (59.83) |
| ADHD S-R clinical diagnosis N (%) | 3 (0.84) | 99 (27.81) |
| Met ASRS cut-off ADHD N (%) | 53 (14.76) | 176 (49.44) |
| Provisional PMDD, N (%) | 50 (13.93) | 95 (26.69) |

Supplemental Table 2: Reasons for exclusion and number of excluded individuals amongst those prescreening positively and negatively for ADHD

|  | Prescreened positive for ADHD  (N=370) | Prescreened negative for ADHD  (N=370) |
| --- | --- | --- |
| Excluded due to participant ineligibility |  |  |
| Age >34 years or missing | 1 | 3 |
| Pregnant | 2 | 2 |
| Breastfeeding | 1 |  |
| Excluded due to invalid data |  |  |
| Duration of survey less than 1 minute | 6 | 5 |
| Endorsed taking ADHD medication without positive endorsement of ADHD diagnosis | 4 | 1 |
| After exclusion total (% of pre-exclusion total) | 356 (96.22) | 359 (97.03) |

*Note: Participants who had ADHD medication without a clinical diagnosis were excluded as their ADHD case status was unclear; presumably ADHD medication use could have been illicit use, but were excluded due to potentially calling into question the presence of an ADHD clinical diagnosis*

Supplemental Table 3: Sociodemographic and clinical characteristics of participants by self-report ADHD and anxiety/depression clinical diagnosis status, and whether they met ASRS-based criteria for ADHD.

|  | No diagnosis reference group (n=187) | Self-reported ADHD clinical diagnosis only (n=25) | Self-reported ADHD and  depression/anxiety diagnosis (n=77) | ASRS-based  ADHD only (n=56) | ASRS-based  ADHD and depression/anxiety diagnosis (n=173) |
| --- | --- | --- | --- | --- | --- |
| Age, mean (SD) | 28.00 (4.03) | 26.4 (4.1) | 28.17 (3.81) | 26.96 (3.78) | 28.25 (3.95) |
| Education, N (%) |  |  |  |  |  |
| Secondary | 108 (35.41) | 9 (36.00) | 20 (25.97) | 15 (26.79) | 49 (28.32) |
| Bachelor's degree | 116 (38.03) | 9 (36.00) | 32 (41.56) | 26 (46.43) | 71 (41.04) |
| Master's degree/PhD | 68 (22.30) | 5 (20.00) | 20 (25.97) | 10 (17.86) | 40 (23.12) |
| Other | 13 (4.26) | 2 (8.00) | 5 (6.49) | 5 (8.93) | 13 (7.51) |
| Hormonal contraceptive use, N (%) | 122 (40.00) | 10 (40.00) | 33 (42.86) | 21 (37.50) | 65 (37.57) |
| Currently taking ADHD medication, N (%) | 0 | 9 (36.00) | 30 (38.96) | 4 (7.14) | 25 (14.45) |
| Ever taken ADHD medication, N (%) | 0 | 19 (76.00) | 46 (59.74) | 11 (19.64) | 35 (20.23) |
| ASRS total score, mean (SD) | 4.52 (3.52) | 11.96 (3.35) | 14.06 (2.98) | 12.58 (3.00) | 13.48 (2.88) |
| Provisional PMDD, N (%) | 30 (9.84) | 5 (20.00) | 27 (35.06) | 17 (30.36) | 77 (44.51) |

Supplemental table 4: Rates of ADHD, depression, and anxiety diagnosis, and PMDD by whether participant endorsed taking hormonal contraceptives.

|  | Taking a hormonal contraceptive (N=282) | |  | Not taking a hormonal contraceptive (N=433) | |
| --- | --- | --- | --- | --- | --- |
|  | N | % |  | N | % |
| Self-reported ADHD clinical diagnosis | 43 | 15.25 |  | 59 | 13.63 |
| ASRS-based ADHD | 86 | 30.5 |  | 143 | 33.03 |
| Self-reported depression diagnosis | 124 | 43.97 |  | 173 | 39.95 |
| Self-reported anxiety diagnosis | 142 | 50.35 |  | 205 | 47.34 |
| Provisional PMDD | 50 | 17.73 |  | 95 | 21.94 |

Hormonal contraceptives include oral hormonal contraceptives, hormonal intrauterine devices, hormonal injections, hormonal implants, and hormonal patches.
