# Supplementary material for: Increased risk of provisional premenstrual dysphoric disorder (PMDD) among females with attention-deficit hyperactivity disorder (ADHD): A cross-sectional survey study
Source: Br J Psychiatry. Author manuscript; Available in PMC 2025 Jun 23. (PMC7617793; doi:10.1192/bjp.2025.104)
Supplement: Supplementary file 1 [file EMS204258-supplement-Supplementary_Materials.pdf]

Supplemental tables and figures

Supplemental Table 1: Sociodemographic and clinical characteristics of individuals who prescreened positively for ADHD and negatively for ADHD included in the current study

	**Prescreened negative for ADHD (N=359)**	**Prescreened positive for ADHD (N=356)**
Age, mean (SD)	28.04 (3.96)	27.75 (3.96)
Education, N (%)		
Secondary	123 (34.26)	99 (27.81)
Bachelor's degree	138 (38.44)	154 (43.26)
Master's degree/PhD	82 (22.84)	77 (21.63)
Other	16 (4.46)	26 (7.30)
Hormonal contraceptive use, N (%)	144 (40.11)	138 (38.76)
Currently taking ADHD medication, N (%)	-	39 (10.96)
Ever taken ADHD medication, N (%)	3 (0.84)	62 (17.42)
ASRS total score (0-18), M (SD)	5.65 (4.40)	11.50 (4.00)
Depression diagnosis, N, (%)	107 (29.81)	190 (53.37)
Anxiety diagnosis, N (%)	134 (37.33)	213 (59.83)
ADHD S-R clinical diagnosis N (%)	3 (0.84)	99 (27.81)
Met ASRS cut-off ADHD N (%)	53 (14.76)	176 (49.44)
Provisional PMDD, N (%)	50 (13.93)	95 (26.69)

Supplemental Table 2: Reasons for exclusion and number of excluded individuals amongst those prescreening positively and negatively for ADHD

	Prescreened positive for ADHD (N=370)	Prescreened negative for ADHD (N=370)
Excluded due to participant ineligibility		
Age >34 years or missing	1	3
Pregnant	2	2
Breastfeeding	1	
Excluded due to invalid data		
Duration of survey less than 1 minute	6	5
Endorsed taking ADHD medication without positive endorsement of ADHD diagnosis	4	1
After exclusion total (% of pre-exclusion total)	356 (96.22)	359 (97.03)

Note: Participants who had ADHD medication without a clinical diagnosis were excluded as their ADHD case status was unclear; presumably ADHD medication use could have been illicit use, but were excluded due to potentially calling into question the presence of an ADHD clinical diagnosis

Supplemental Table 3: Sociodemographic and clinical characteristics of participants by self-report ADHD and anxiety/depression clinical diagnosis status, and whether they met ASRS-based criteria for ADHD.

	No diagnosis reference group (n=187)	Self-reported ADHD clinical diagnosis only (n=25)	Self-reported ADHD and depression/anxiety diagnosis (n=77)	ASRS-based ADHD only (n=56)	ASRS-based ADHD and depression/anxiety diagnosis (n=173)
Age, mean (SD)	28.00 (4.03)	26.4 (4.1)	28.17 (3.81)	26.96 (3.78)	28.25 (3.95)
Education, N (%)					
Secondary	108 (35.41)	9 (36.00)	20 (25.97)	15 (26.79)	49 (28.32)
Bachelor's degree	116 (38.03)	9 (36.00)	32 (41.56)	26 (46.43)	71 (41.04)
Master's degree/PhD	68 (22.30)	5 (20.00)	20 (25.97)	10 (17.86)	40 (23.12)
Other	13 (4.26)	2 (8.00)	5 (6.49)	5 (8.93)	13 (7.51)
Hormonal contraceptive use, N (%)	122 (40.00)	10 (40.00)	33 (42.86)	21 (37.50)	65 (37.57)
Currently taking ADHD medication, N (%)	0	9 (36.00)	30 (38.96)	4 (7.14)	25 (14.45)
Ever taken ADHD medication, N (%)	0	19 (76.00)	46 (59.74)	11 (19.64)	35 (20.23)
ASRS total score, mean (SD)	4.52 (3.52)	11.96 (3.35)	14.06 (2.98)	12.58 (3.00)	13.48 (2.88)
Provisional PMDD, N (%)	30 (9.84)	5 (20.00)	27 (35.06)	17 (30.36)	77 (44.51)

Supplemental table 4: Rates of ADHD, depression, and anxiety diagnosis, and PMDD by whether participant endorsed taking hormonal contraceptives.

	Taking a hormonal contraceptive (N=282)		Not taking a hormonal contraceptive (N=433)	
	N	%	N	%
Self-reported ADHD clinical diagnosis	43	15.25	59	13.63
ASRS-based ADHD	86	30.5	143	33.03
Self-reported depression diagnosis	124	43.97	173	39.95
Self-reported anxiety diagnosis	142	50.35	205	47.34
Provisional PMDD	50	17.73	95	21.94

Hormonal contraceptives include oral hormonal contraceptives, hormonal intrauterine devices, hormonal injections, hormonal implants, and hormonal patches.

Methods supplement

Appendix S1: Questionnaire Appendix

Demographic Questions

What is your age? ____

What is the highest level of education you've attained?

- Secondary school[a]
- Bachelor's degree
- Master's degree
- PhD
- Other (please specify________)

Other questions

Are you currently pregnant?

- Yes
- No

Are you currently breastfeeding?

- Yes
- No

Medication Questions

Do you take any medication for ADHD?

If so, what medication ______________

Have you ever taken any medication for ADHD?

Do you use any of the following contraceptives?

- Birth control pills
- The hormonal coil (or hormonal IUD; e.g. Mirena coil)[b]
- The copper coil (or non-hormonal IUD) [b]
- Other (please specify________)

Has a clinician ever diagnosed you with any of the following:

- ADHD
- Depression
- Anxiety
- None of the above

Appendix S2 – Further questionnaire information

Adult ADHD Self-Report Scale (ASRS)

The ASRS includes eighteen items based on DSM-IV-TR ADHD criteria rated on a five-point scale (“Never”, “Rarely” “Sometimes”, “Often”, “Very Often”); an item was considered to be positively endorsed based on recommended cut-offs. Population surveys have shown that the ASRS has a moderate sensitivity (68.7%), high specificity (99.5%(1)), high internal consistency(2) and good test-retest reliability.(3)

Premenstrual symptoms screening tool (PSST)

The PSST operationalises DSM-IV-TR criteria for PMDD and queries whether females experience symptoms that start in the days prior to their period and stop within a few days of bleeding (4). Fourteen items related to premenstrual symptoms (as highlighted in the introduction) are included. The impact of symptoms on functioning in work/school, social, and family settings was also measured; each item is rated on a four-point scale (‘Not at all’, ‘Mild’, ‘Moderate’, ‘Severe’). In order to meet criteria for provisional PMDD: (a) at least one of the first four items (‘Anger/irritability’; ‘Anxiety/tension’ ‘Tearful/increased sensitivity to rejection’; ‘Depressed mood/hopelessness’) must be rated as severe, (b) at least four of the other fourteen items must be rated as moderate to severe, and (c) impairment must be rated as severe in at least one setting.

Refences

1. Kessler RC, Adler L, Ames M, Demler O, Faraone S, Hiripi E, et al. The World Health Organization Adult ADHD Self-Report Scale (ASRS): a short screening scale for use in the general population. Psychol Med. 2005;35(2):245-56.

2. Adler LA, Spencer T, Faraone SV, Kessler RC, Howes MJ, Biederman J, Secnik K. Validity of pilot Adult ADHD Self- Report Scale (ASRS) to Rate Adult ADHD symptoms. Ann Clin Psychiatry. 2006;18(3):145-8.

3. Matza LS, Van Brunt DL, Cates C, Murray LT. Test-retest reliability of two patient-report measures for use in adults with ADHD. J Atten Disord. 2011;15(7):557-63.

4. Steiner M, Macdougall M, Brown E. The premenstrual symptoms screening tool (PSST) for clinicians. Arch Womens Ment Health. 2003;6(3):203-9.
